# Social distribution of tobacco smoking, alcohol drinking and obesity in the French West Indies

**DOI:** 10.1186/s12889-019-7802-1

**Published:** 2019-10-30

**Authors:** Aviane Auguste, Julien Dugas, Gwenn Menvielle, Christine Barul, Jean-Baptiste Richard, Danièle Luce

**Affiliations:** 1 Univ Rennes, Inserm, EHESP, Irset (Institut de recherche en santé, environnement et travail) -UMR_S 1085, F-97100 Pointe-à-Pitre, France; 2 Sorbonne Université, INSERM, Institut Pierre Louis d’Epidémiologie et de Santé Publique (IPLESP), F75012 Paris, France; 3 Santé publique France, French National Public Health Agency, 12 rue du val d’Osne, F-94415 Saint Maurice, France

**Keywords:** Social disparities, Tobacco smoking, Alcohol drinking, Obesity, Non-communicable diseases, Caribbean, France

## Abstract

**Background:**

Tobacco smoking, alcohol and obesity are important risk factors for a number of non-communicable diseases. The prevalence of these risk factors differ by socioeconomic group in most populations, but this socially stratified distribution may depend on the social and cultural context. Little information on this topic is currently available in the Caribbean. The aim of this study was to describe the distribution of tobacco smoking, alcohol drinking and obesity by several socioeconomic determinants in the French West Indies (FWI).

**Methods:**

We used data from a cross-sectional health survey conducted in Guadeloupe and Martinique in 2014 in a representative sample of the population aged 15–75 years (*n* = 4054). All analyses were stratified by gender, and encompassed sample weights, calculated to account for the sampling design and correct for non-response. For each risk factor, we calculated weighted prevalence by income, educational level, occupational class and having hot water at home. Poisson regression models were used to estimate age-adjusted prevalence ratios (PR) and 95% confidence intervals (CI).

**Results:**

Current smoking and harmful chronic alcohol use were more common in men than in women (PR = 1.80, 95% CI = 1.55–2.09; PR = 4.53, 95% CI = 3.38–6.09 respectively). On the other hand, the prevalence of obesity was higher in women than in men (PR = 0.67, 95% CI = 0.57–0.79). Higher education, higher occupational class and higher income were associated with lower prevalence of harmful alcohol drinking in men (PR = 0.43, 95% CI = 0.25–0.72; PR = 0.73, 95% CI = 0.53–1.01; PR = 0.72, 95% CI = 0.51–1.03 respectively), but not in women. For tobacco smoking, no variation by socioeconomic status was observed in men whereas the prevalence of current smoking was higher among women with higher occupational class (PR = 1.47, 95% CI = 1.13–1.91) and higher income (PR = 1.50, 95% CI = 1.11–2.03). In women, a lower prevalence of obesity was associated with a higher income (PR = 0.43, 95% CI = 0.33–0.56), a higher occupational class (PR = 0.63, 95% CI = 0.50–0.80), a higher educational level (PR = 0.36, 95% CI = 0.26–0.50) and having hot water at home (PR = 0.65, 95% CI = 0.54–0.80).

**Conclusion:**

Women of high socio-economic status were significantly more likely to be smokers, whereas alcohol drinking in men and obesity in women were inversely associated with socioeconomic status.

## Background

The French West Indies (FWI) is a part of the Caribbean region which is made up of the two overseas French regions, Martinique and Guadeloupe. The French West Indies have a particular situation in the Caribbean. As French territories, Martinique and Guadeloupe are classified as high-income countries, whereas most of other Caribbean states are low or middle-income countries. The FWI population benefits from the same health insurance and financial redistribution systems as the mainland French population. While the French West Indies appear to be a privileged region within the Caribbean, the comparison with the mainland is much less favourable. Although the gross domestic product per capita is one of the highest in the Caribbean, it is only about 65% of the French national average. When compared to the national average, the population of the FWI is characterized by a lower median income, a lower educational level and a higher rate of unemployment. On the other hand, the FWI are close to their Caribbean neighbours with regards to the cultural, historical and climatic context. This unique situation reflects in health conditions, with for most of them an intermediate position between mainland France and other countries in the Caribbean. Cancer and cardiovascular diseases were in 2016 the leading causes of death in the FWI, accounting each for about 25% of all deaths [1]. Cancer incidence rates are overall lower than in mainland France, with the exception of prostate, stomach and cervical cancer, but higher than in other Caribbean countries for most cancer sites. Mortality rates from cardiovascular diseases, although higher than in mainland France, are among the lowest in the Caribbean [1–5]. The prevalence of diabetes is also high in the FWI [6]. Tobacco smoking, alcohol drinking and obesity are important risk factors for a number of non-communicable diseases (NCD), including cancer, diabetes and cardiovascular disease. These risk factors were described in previous studies to be inequitably distributed across the different socio-economic strata. Worldwide, the prevalence of these risk factors tends to be higher in persons of lower socioeconomic status (SES) than in the more affluent groups [7, 8]. This trend however varies with country-level development and the indicators used [7–10]; in mainland France, and other developed countries, lower SES is usually associated to a greater prevalence of these risk factors; whereas, in low and middle-income countries, the reverse association is usually observed [7, 11–13]. However, data in regards to social disparities and NCD risk factors are very scarce in the Caribbean. A study in Barbados addressed the social distribution of NCD risk factors [14]. A systematic review reported data on social determinants of obesity and alcohol consumption in the Caribbean; however, they provide unclear conclusions on the social disparities in this population, due to few data [15]. Knowing the social distribution of risk factors is crucial for the designing of prevention programs and policy in these regions [15]. The specific features of the FWI further warrant a sound understanding of the social distribution of the known NCD risk factors to take appropriate measures for prevention.

In this study, we performed a secondary data analysis from a national survey in order to describe the social distribution of tobacco smoking, alcohol drinking and obesity in the French West Indies.

## Methods

### Study population, data collection

The data for this study were drawn from a national cross-sectional health survey conducted in the FWI in 2014 (“Baromètre Santé DOM”, Health Barometer) [16]. The survey was based on a random two stage sampling method: telephone numbers (landlines and cell phones) were randomly generated, then one person was randomly selected among eligible household members or among cell phone users, using the Kish method [17]. Persons aged between 15 and 75 years of age living in Martinique or Guadeloupe who spoke French or Creole were eligible for inclusion. Field investigators conducted the interview over the phone.

Participation was anonymous and voluntary. Anonymity and respect of confidentiality were guaranteed using a procedure erasing the phone number. All included subjects gave informed consent before the telephone interview. Parental consent was obtained for participants under 18. As the participants were contacted exclusively over the phone, the consents were verbal. The overall procedure was approved by the French regulatory authority, the Commission Nationale de l’Informatique et des Libertés (CNIL).

Overall, 8057 numbers were dialled (3687 landlines and 4407 cell phones). Among them, 35% could not be reached, 11% refused to participate and 3% abandoned the survey before the end of the interview. In the end, 4054 subjects were included in the final sample for Martinique and Guadeloupe. The overall participation rate for the French West Indies was 51% (56% for landlines, 46% for cell phones).

Data were weighted in two steps. To account for the sampling design, sample weights were computed according to the probability of selection of the telephone number, the number of eligible individuals for each telephone number, the number of landline and cell phones of the individual. To correct for non-response, a post-stratification was then performed to match the distribution of the population, according to sex, age, education level and household structure, using data from the 2011 census in Martinique and Guadeloupe.

### Variables

All risk factors analysed in our current study were dichotomised. Current smokers were persons who smoked any tobacco product. Lifetime tobacco smokers were those who had smoked tobacco in their lifetime regardless of the duration or frequency. Daily alcohol drinkers were persons who drank at least one glass of alcohol per day. Harmful chronic alcohol use was defined as drinking more than 21 drinks a week for a man and 14 for a woman or drinking six drinks or more on a single occasion weekly [18]. Self-reported height and weight were collected during the phone call and body mass index (BMI) was calculated (weight in kg/height in m^2^). An obese person was regarded as someone with a BMI of at least 30 kg/m^2^. We used four variables related to socioeconomic status: education, occupational category, income and having hot water at home. Education was defined as the highest educational attainment achieved by an individual participant and categorised into four groups: without diploma or primary education (up to approximately 6 years of schooling), less than high school diploma (up to approximately 9 years of schooling), high school diploma (up to approximately 12 years), and tertiary education (associate’s degree or higher) [19]. Occupation was defined as the current occupation for active workers and as the last occupation for retired or unemployed persons, and was classified into three groups based on the French classification of occupations and socio-professional categories [20, 21]: qualified workers (self-employed and entrepreneurs, professionals and managers), unqualified workers (farmers, clerical, sales and service workers, manual workers) and inactive, who were persons who never worked.. Individual income was split into three groups according to the tertiles of the overall distribution of income in our sample. Having hot water at home described someone living in a household where a water heating system was available to heat the running water in the house. Hot water at home is strongly linked to the household income in the FWI and can therefore be viewed as a surrogate for self-reported income, which may be more subject to misclassification or misreporting [22].

### Statistical analysis

The prevalence for each risk factor was calculated by gender, age and according to the four socio-economic indicators. Age-adjusted prevalence ratios (PR) and their 95% confidence intervals (CI) estimating the associations of the different socio-economic indicators with the risk factors were calculated using a Poisson-regression model. Chi-squared tests were performed to assess the statistical trend between the socio-demographics and gender. All analyses encompassed sample weights.

## Results

### Characteristics and risk factor prevalence

In total 4054 persons were included for the purpose of our analysis. Table 1 shows the distribution of socio-demographic characteristics of participants in our sample. The participants were equally distributed between Martinique and Guadeloupe and there were slightly more women than men (ratio of women to men 1.2). Men were more frequently under 25 years of age and had higher income when compared to women. On the other hand, women had more frequently tertiary education and hot water at home when compared to men. Very few data were missing for most variables (≤1%) with the exception of individual income and body mass index (14 and 6% respectively). Table 2 shows the prevalence of risk factors. Overall, ever tobacco smoking was the most prevalent risk factor among participants. Men were significantly more likely to be smokers and alcohol drinkers. The prevalence of ever and current smokers was two-fold grater in men than in women (PR = 1.98, 95% CI = 1.75–2.24 and PR = 1.80, 95% CI = 1.55–2.09 respectively). Similarly, the prevalence of daily alcohol drinking and harmful chronic drinking was 4 times greater in men than in women (PR = 4.15, 95% CI = 3.11–5.55 and PR = 4.53, 95% CI = 3.38–6.09 respectively). Inversely men were significantly less likely to be obese than women (PR = 0.67, 95% CI = 0.57–0.79).

**Table 1:** Distribution of socio-demographic characteristics of participants by gender

Characteristic	Category	Men		Women		Overall		*p* ^†^
		*n* = 1849	%^a^	*n* = 2205	%^a^	*n* = 4054	% ^a^	
Age (years)								0.0721
	15–24	351	(19.0)	348	(15.8)	699	(17.2)	
	25–34	232	(12.5)	311	(14.1)	543	(13.4)	
	35–44	348	(18.8)	459	(20.8)	807	(19.9)	
	45–54	396	(21.4)	466	(21.1)	862	(21.3)	
	55–64	303	(16.4)	359	(16.3)	662	(16.3)	
	65–75	219	(11.8)	263	(11.9)	481	(11.9)	
Recruitement site								0.9135
	Martinique	922	(49.9)	1104	(50.1)	2026	(50.0)	
	Guadeloupe	927	(50.1)	1101	(49.9)	2028	(50.0)	
Education level								< 0.0001
	Up to primary education	462	(25.2)	511	(23.3)	973	(24.2)	
	Less than high school diploma	811	(44.3)	818	(37.4)	1629	(40.5)	
	High school diploma	279	(15.2)	421	(19.2)	700	(17.4)	
	Tertiary education	280	(15.3)	439	(20.1)	719	(17.9)	
	Missing	17		16		33		
Occupational Class								0.0567
	Inactive	245	(13.2)	339	(15.4)	584	(14.4)	
	Non-qualified	1032	(55.9)	1241	(56.4)	2273	(56.1)	
	Qualified	571	(30.9)	621	(28.2)	1191	(29.4)	
	Missing	1		4		5		
Individual income								< 0.0001
	Low-income	471	(30.3)	724	(37.7)	1195	(34.4)	
	Middle-income	523	(33.7)	630	(32.8)	1153	(33.2)	
	High-income	561	(36.1)	566	(29.5)	1127	(32.4)	
	Missing	294		285		579		
Hot water at home								0.0153
	Yes	1283	(69.5)	1609	(73.0)	2892	(71.4)	
	No	563	(30.5)	596	(27.0)	1159	(28.6)	
	Missing	3		0		3		

Baromètre Santé DOM survey, 2014

^a^Column percentage calculated by dividing the total number of men,women or overall sample

†: *p*-value of Chi-squared test, assessing the association between participant’s socio-demographic characteristics and gender

**Table 2:** Prevalence of risk factors and prevalence ratios comparing men to women

Risk factor	Men		Women	
	*n* = 1849		*n* = 2205	
Ever tobacco smoking				
Prevalence (%)	682	(38)	421	(19)
Adjusted PR (95% CI)	1.98	(1.75–2.24)	1	ref
Missing (n)	36		8	
Current tobacco smoking				
Prevalence (%)	422	(23)	286	(13)
Adjusted PR (95% CI)	1.80	(1.55–2.09)	1	ref
Missing (n)	43		0	
Daily alcohol drinking				
Prevalence (%)	201	(11)	59	(3)
Adjusted PR (95% CI)	4.15	(3.11–5.55)	1	ref
Missing (n)	1		0	
Harmful chronic alcohol use				
Prevalence (%)	213	(12)	57	(3)
Adjusted PR (95% CI)	4.53	(3.38–6.09)	1	ref
Missing (n)	1		0	
Obesity				
Prevalence (%)	209	(12)	443	(22)
Adjusted PR (95% CI)	0.67	(0.57–0.79)	1	ref
Missing (n)	100		146	

*PR* Prevalence ratio, *95%CI* 95% confidence interval

Baromètre Santé DOM survey, 2014

Table 3 shows the prevalence of risk factors by gender and age. In both men and women, for all tobacco and alcohol-related variables, the highest prevalence was consistently observed in the 25 to 34 age group when compared to the other age groups. We observed a regular decrease of the prevalence of current tobacco smoking from 24 to 75 years of age, A similar trend, although less apparent, was found for ever smoking. On the other hand, in both men and women, daily alcohol drinking increased with age whereas harmful chronic alcohol drinking decreased with age. In terms of obesity, women between 55 and 64 years were the most frequently obese (28.9%), followed by the 25 to 34 age group with 23.8%. The obesity prevalence in men was quite homogenous across age groups with the exception of men under 24 years for whom the prevalence was notably lower (4.9%).

**Table 3:** Risk factor prevalence by age group

Risk factor	15–24 yrs		25–34 yrs		35–44 yrs		45–54 yrs		55–64 yrs		65–75 yrs	
	*n* = 699	%	*n* = 543	%	*n* = 807	%	*n* = 862	%	*n* = 662	%	*n* = 481	%
Ever tobacco smoking												
Total	180	(25.8)	191	(35.2)	224	(27.7)	227	(26.4)	174	(26.2)	107	(22.3)
Women	72	(20.9)	87	(28.4)	101	(22.0)	90	(19.3)	48	(13.3)	23	(8.8)
Men	108	(32.4)	104	(46.5)	123	(35.7)	137	(35.1)	126	(41.7)	84	(38.6)
Current tobacco smoking												
Total	167	(24.5)	155	(29.2)	153	(19.1)	133	(15.5)	68	(10.2)	33	(6.9)
Women	64	(18.6)	69	(22.6)	69	(15.0)	65	(14.0)	13	(3.6)	6	(2.4)
Men	102	(30.6)	86	(38.4)	84	(24.6)	68	(17.2)	55	(18.2)	27	(12.3)
Daily alcohol drinking												
Total	17	(2.4)	41	(7.6)	41	(5.1)	43	(5.0)	63	(9.5)	55	(11.4)
Women	1	(0.2)	14	(4.4)	15	(3.3)	7	(1.4)	9	(2.6)	13	(5.1)
Men	16	(4.6)	27	(11.8)	26	(7.5)	37	(9.3)	54	(17.7)	41	(18.9)
Harmful chronic alcohol use												
Total	73	(10.4)	60	(11.1)	56	(7.0)	44	(5.1)	23	(3.4)	13	(2.8)
Women	15	(4.4)	14	(4.5)	13	(2.9)	10	(2.1)	1	(0.2)	3	(1.3)
Men	58	(16.4)	46	(20.0)	43	(12.3)	34	(8.6)	22	(7.3)	10	(4.6)
Obesity												
Total	51	(7.9)	96	(19.0)	130	(17.1)	159	(19.6)	139	(21.8)	78	(17.0)
Women	35	(11.0)	70	(23.8)	83	(19.6)	103	(23.4)	101	(28.9)	52	(21.7)
Men	16	(4.9)	26	(12.3)	47	(13.9)	56	(15.1)	38	(13.3)	26	(11.9)

Baromètre Santé DOM survey, 2014

### Social distribution of risk factors

Tables 4 and 5 show in women and men respectively, the prevalence of risk factors by socio-economic category, as well as age-adjusted prevalence ratios, and 95% CI of the Poisson regression model, estimating the associations between the socio-economic indicators and those risk factors. In women, ever smoking prevalence was seen to increase with higher socio-economic status. The prevalence was significantly greater in women who had tertiary education (PR = 1.45, 95% CI = 1.07–1.96), and who occupied qualified jobs (PR = 1.60, 95% CI = 1.30–1.98) and who had the highest incomes (PR = 1.63, 95% CI = 1.28–2.08). Similarly, compared to persons in lower SES class, current smoking prevalence was significantly greater in women of in qualified jobs, and those had higher income. In contrast, daily alcohol and harmful chronic alcohol drinking were not associated with SES in women. However, though the prevalence difference for occupational class was not significant, women with qualified jobs, and hot water at home tended to engage less in harmful chronic drinking. Having hot water at home was not significantly associated with tobacco and daily alcohol consumption. In men, no distinct trend or significant association was found in regards to tobacco and socio-economic status. However, in men, a harmful chronic drinking and daily alcohol drinking were inversely and significantly associated with educational level With the exception of daily alcohol in women, we found that occupationally inactive persons had significantly lower alcohol drinking prevalence for both genders when compared to unqualified workers. Obesity prevalence was inversely associated with socio-economic status, in particular in women, where we observed significant decreases of at least 35% in obesity prevalence in those of the highest stratum for each socio-economic indicator (education PR = 0.36, 95% CI = 0.26–0.50; occupational class PR = 0.63, 95% CI = 0.50–0.80; income PR = 0.43, 95% CI = 0.33–0.56; hot water at home PR = 0.65, 95% CI = 0.54–0.80).

**Table 4:** Associations between SES and risk factors in women

SES indicator	Ever tobacco		Current tobacco		Daily alcohol		Harmful chronic alcohol use		Obesity	
	Prev	PR (95% CI)	Prev	PR (95% CI)	Prev	PR (95% CI)	Prev	PR (95% CI)	Prev	PR (95% CI)
Education level										
Up to primary education	14.8	1 (ref)	10.4	1 (ref)	2.9	1 (ref)	2.7	1 (ref)	31.3	1 (ref)
Less than high school diploma	16.2	1.00 (0.75–1.33)	11.1	0.89 (0.63–1.25)	3.2	1.29 (0.67–2.48)	1.9	0.55 (0.26–1.16)	24.5	0.79 (0.63–0.98)
High school diploma	23.2	1.30 (0.96–1.78)	16.8	1.13 (0.79–1.63)	1.9	1.71 (0.68–4.28)	4.0	0.97 (0.46–2.02)	14.9	0.53 (0.39–0.73)
Tertiary education	26.2	1.45 (1.07–1.96)	16.5	1.09 (0.76–1.58)	2.2	0.72 (0.30–1.70)	2.3	0.50 (0.21–1.19)	12.1	0.36 (0.26–0.50)
Occupational Class										
Inactive	18.9	1.04 (0.73–1.49)	16.4	1.02 (0.68–1.53)	0.7	1.19 (0.35–4.04)	1.6	0.21 (0.08–0.55)	17.2	1.27 (0.88–1.83)
Non-qualified	15.6	1 (ref)	10.3	1 (ref)	3.0	1 (ref)	3.1	1 (ref)	25.4	1 (ref)
Qualified	26.3	1.60 (1.30–1.98)	16.7	1.47 (1.13–1.91)	3.2	1.07 (0.61–1.85)	2.0	0.54 (0.27–1.05)	16.2	0.63 (0.50–0.80)
Individual income										
Low-income	16.6	1 (ref)	11.6	1 (ref)	2.5	1 (ref)	2.5	1 (ref)	31.8	1 (ref)
Middle-income	17.6	1.12 (0.86–1.45)	13.3	1.21 (0.89–1.63)	2.4	0.87 (0.43–1.76)	3.0	1.14 (0.60–2.18)	19.2	0.59 (0.47–0.74)
High-income	25.5	1.63 (1.28–2.08)	15.6	1.50 (1.11–2.03)	3.7	1.38 (0.74–2.61)	3.0	1.22 (0.63–2.38)	14.3	0.43 (0.33–0.56)
Hot water at home										
Yes	19.3	1.06 (0.85–1.32)	13.1	1.06 (0.81–1.37)	2.6	0.76 (0.40–1.30)	2.1	0.60 (0.35–1.02)	19.0	0.65 (0.54–0.80)
No	18.5	1 (ref)	13.0	1 (ref)	2.9	1 (ref)	3.8	1 (ref)	28.6	1 (ref)

*Prev* Risk factor prevalence, *PR* Age-adjusted prevalence ratio, *95%CI* 95% confidence interval

Baromètre Santé DOM survey, 2014

**Table 5:** Associations between SES and risk factors in men

SES indicator	Ever tobacco		Current tobacco		Daily alcohol		Harmful chronic alcohol use		Obesity	
	Prev	PR (95% CI)	Prev	PR (95% CI)	Prev	PR (95% CI)	Prev	PR (95% CI)	Prev	PR (95% CI)
Education level										
Up to primary education	40.3	1 (ref)	22.9	1 (ref)	15.2	1 (ref)	13.2	1 (ref)	15.2	1 (ref)
Less than high school diploma	36.2	0.91 (0.75–1.09)	22.6	0.91 (0.72–1.17)	12.0	0.87 (0.64–1.18)	12.1	0.82 (0.59–1.13)	11.4	0.79 (0.57–1.10)
High school diploma	35.9	0.89 (0.69–1.14)	26.2	0.88 (0.65–1.21)	6.0	0.46 (0.26–0.80)	12.7	0.74 (0.48–1.15)	9.7	0.77 (0.48–1.22)
Tertiary education	40.1	0.98 (0.77–1.25)	23.9	0.90 (0.66–1.23)	5.5	0.40 (0.23–0.70)	6.8	0.43 (0.25–0.72)	10.0	0.64 (0.40–1.01)
Occupational Class										
Inactive	27.1	0.67 (0.48–0.94)	24.5	0.63 (0.44–0.91)	2.4	0.31 (0.12–0.81)	9.9	0.37 (0.22–0.62)	5.2	1.11 (0.43–2.87)
Non-qualified	38.6	1 (ref)	24.1	1 (ref)	12.2	1 (ref)	13.4	1 (ref)	13.4	1 (ref)
Qualified	40.3	1.04 (0.88–1.22)	21.3	0.95 (0.76–1.19)	12.2	0.93 (0.69–1.25)	8.9	0.73 (0.53–1.01)	12.1	0.88 (0.65–1.18)
Individual income										
Low-income	36.1	1 (ref)	24.4	1 (ref)	11.8	1 (ref)	15.1	1 (ref)	13.2	1 (ref)
Middle-income	34.8	0.98 (0.79–1.21)	19.2	0.84 (0.64–1.10)	10.4	0.85 (0.59–1.24)	9.6	0.68 (0.48–0.98)	11.0	0.70 (0.48–1.02)
High-income	42.7	1.18 (0.97–1.44)	24.4	1.08 (0.84–1.40)	9.3	0.74 (0.50–1.08)	10.0	0.72 (0.51–1.03)	12.1	0.76 (0.52–1.09)
Hot water at home										
Yes	38.1	1.02 (0.87–1.21)	23.5	1.02 (0.83–1.26)	10.8	0.94 (0.69–1.26)	10.0	0.68 (0.52–0.89)	10.4	0.65 (0.49–0.87)
No	36.8	1 (ref)	22.9	1 (ref)	11.2	1 (ref)	15.0	1 (ref)	15.6	1 (ref)

*Prev* Risk factor prevalence, *PR* Age-adjusted prevalence ratio, *95%CI* 95% confidence interval

Baromètre Santé DOM survey, 2014

## Discussion

Social disparities in NCD risk factors distribution were reported in previous studies in many countries [7, 14, 15, 23] but data on this topic are scarce in the Caribbean. We attempted to shed some light on disparities in chronic diseases by describing the social distribution of these risk factors in the French West Indies. We were able to highlight gender-specific social disparities in regards to these risk factors, in this population.

While tobacco smoking was predominantly found in women of high SES, in men, the prevalence did not differ in regards to SES. The social pattern for tobacco smoking did not correspond to what has been described in developed countries, and in particular in mainland France, where persons of lower SES were more frequently smokers [24–26]. Furthermore, the social distribution of tobacco smoking in Barbados and Cuba was discordant with what we found. In men, a negative association between smoking and SES was found in both countries. In women, the social distribution for tobacco smoking in Barbados did not have any distinct pattern and in Cuba it went in the opposite direction to ours [14, 27]. Previous reports have shown that economic development and urbanicity affect socio-economic behaviour and would explain the variation of our results from other studies [7, 8]. Data from the World Health Surveys in 53 countries showed that in the most urban countries, which were mainly middle-income countries in this study, smoking in women was concentrated in the higher education groups, whereas in men smoking was inversely associated with education, regardless of urbanicity [7]. The FWI have a high level of urbanicity, with more than 80% of the population living in urban areas, and our results are consistent with these findings for women, but not for men. The global tobacco epidemic, as described elsewhere, explains well these differences [26]. It is a process which begins first in the most affluent men in society; then, it spreads through the other socioeconomic classes. The same habit then initiates in women of high SES; before finally transitioning to the lower socioeconomic class, since those in higher SES tend to become conscious of their unhealthy lifestyle and possess greater means to alter their behaviour or environment. Our findings suggest that the FWI have not reached the last stage of tobacco epidemic, and that tobacco consumption could increase in the lower SES categories in the future.

The association between alcohol use and SES is complex, vary across genders, country development level and cultures, and depends on the measures used for alcohol drinking [12, 28]. Alcohol drinking measures differ in the previous studies, which made comparisons difficult. We found that in men the prevalence of daily alcohol drinking and harmful alcohol use was lower in the highest socioeconomic strata, a pattern consistent with the inverse association with SES reported in Barbados for heavy episodic alcohol consumption [14] and in a multinational study (including France) for heavy drinking [12]. In women, no clear trend was found, similarly to Barbados [14] but inconsistent with mainland France where the prevalence of heavy drinking was higher in the highest educational level [12].

In terms of obesity, there was an inverse association with the socio-economic status for both genders with a more marked socioeconomic gradient in women. This gradient was the most apparent for income, where the prevalence was twice as high in women of low income when compared to those of high income. A French study [29] and a study in Guadeloupe [6] reported a social pattern for obesity in men and women concordant to our sample. In contrast, the study in Barbados reported no socioeconomic gradient for obesity [14]. Previous studies showed that in developed countries, women of high socio-economic status are more sensitive to body image because small body size is viewed as attractive [30, 31]. Although in our study height and weight were self-reported, our findings were globally similar to those of studies that used anthropometric measurements [6, 29].

Our findings are also consistent with the local context. A previous study conducted in the FWI investigating area-level socio-economic status and incidence of cancer revealed that women living in deprived areas were found to have a lower incidence of lung and head and neck cancers when compared to more affluent areas, which is consistent with the lower prevalence of tobacco smoking (a major risk factor for respiratory cancer) among women of low SES reported in our study [32]. That same study showed that breast cancer incidence was higher in women from deprived areas. Our results on obesity, a known risk factor for breast cancer, coincided well with the incidence data in that study, since our female obesity was consistently more prevalent in the lower SES strata.

In addition to the social distribution, our analysis revealed interesting estimates for the prevalence of risk factors by gender. The FWI were found to have a particular NCD risk factor profile, especially when compared to Caribbean neighbours and mainland France. Overall, the prevalence of risk factors in the FWI was in-between mainland France and other Caribbean territories. The prevalence of current smokers was 23% in men and 13% in women, lower than in mainland France (32.3 and 24.3%) [33], and similar in men to the other territories in the Caribbean [4]. However, in the FWI the prevalence of current smokers in women was higher than in other Caribbean territories (5.9% in Jamaica and 3.7% in Barbados) [14, 34]. Daily alcohol drinking prevalence was also lower in the FWI (12% in men, 3% in women) than in mainland France (15% in men, 5% in women) [35]. Harmful chronic alcohol use was however similar in men (FWI: 12%, mainland France: 11%) and in women (FWI: 3%, mainland France: 4%). The other reports in the Caribbean used different definitions for alcohol drinking to us which made it difficult to evaluate differences between countries.

In our study obesity prevalence was assessed from self-reported data and may be underestimated [36]. In a survey in mainland France using the same methodology than ours, obesity prevalence in women (12%) was lower to that in the FWI (22%), whereas in men the prevalence was similar in both territories (12%) [37]. In a survey based on measurements of height and weight conducted in 2008 in the FWI obesity prevalence was slightly higher than in our study (17% in men and 27% in women) [38]. A national survey in mainland France, also using measurements, reported an obesity prevalence of 16% in men and 17% in women [29]. It should be noted that regardless the method used (self-report or measurements): the prevalence of obesity in men is similar in mainland France and in the FWI; the prevalence of obesity in women is higher in the FWI; in mainland France, the prevalence of obesity is similar in men and women, whereas in the FWI obesity is much more frequent in women., On the contrary, obesity among men and women in the FWI was much lower compared to the prevalence reported by Caribbean neighbours and Nicaragua [14, 39–42]. These observed differences could be due to the FWI being overseas French regions, and the population may share similar behaviour patterns from their mainland counterparts; however, they are under the Caribbean influence due to their geographic position. These conditions could explain this particular risk factor profile in the FWI.

Our study presents some limitations that should be taken into consideration when interpreting our results. The socio-economic indicators and risk factors were measured through self-reported data from our study participants and thus, are subject to misclassification bias. We cannot exclude the possibility that this misclassification was related to SES, which may have impacted our results on the social distribution of risk factors. Our study has also several strengths. Our sample size was quite large (4054 participants), and therefore could provide fairly reliable estimates and we corrected for the non-response bias by using sample weights. Our sample was representative of the FWI and included participants from both rural and urban areas; hence, our results can be considered generalisable to the FWI.

## Conclusion

Our analysis revealed gender-specific social disparities in NCD risk factor distribution. Women of high socio-economic status were significantly more likely to be smokers, whereas alcohol drinking in men and obesity in women were inversely associated with socioeconomic status. Future prevention programs and policies should take into consideration our findings.

## Data Availability

Data are from the “Baromètre Santé DOM” carried out by the French National Public Health Agency (Santé Publique France). This analysis has been subject to a license for the use of data issued by Santé Publique France. Readers may contact Jean-Baptiste Richard (jean-baptiste.richard@santepubliquefrance.fr) to request the data.

## Abbreviations

BMI: Body mass index
CI: Confidence interval
FWI: French West Indies
PR: Prevalence ratio
SES: Socioeconomic status
